# Metabolomics Approaches for the Diagnosis, Treatment, and Better Disease Management of Viral Infections

**DOI:** 10.3390/metabo13080948

**Published:** 2023-08-15

**Authors:** Haya Al-Sulaiti, Jehad Almaliti, C. Benjamin Naman, Asmaa A. Al Thani, Hadi M. Yassine

**Affiliations:** 1 QU Health, Qatar University, Doha P.O. Box 2713, Qatar; haya.alsulaiti@qu.edu.qa (H.A.-S.); aaja@qu.edu.qa (A.A.A.T.); 2 Biomedical Research Center, Qatar University, Doha P.O. Box 2713, Qatar; 3 Scripps Institution of Oceanography, Skaggs School of Pharmacy and Pharmaceutical Sciences, University of California San Diego, La Jolla, CA P.O. Box 92093, USA; jalmaliti@ucsd.edu; 4 Department of Pharmaceutical Sciences, College of Pharmacy, The University of Jordan, Amman P.O. Box 11942, Jordan; 5 Department of Science and Conservation, San Diego Botanic Garden, Encinitas, CA P.O. Box 92024, USA; bnaman@sdbgarden.org; 6 College of Health Sciences, QU-Health, Qatar University, Doha P.O. Box 2713, Qatar

**Keywords:** metabolomics, NMR, LC-MS, viral infections, COVID-19, HIV, HCV, HBV, HCMV, influenza

## Abstract

Metabolomics is an analytical approach that involves profiling and comparing the metabolites present in biological samples. This scoping review article offers an overview of current metabolomics approaches and their utilization in evaluating metabolic changes in biological fluids that occur in response to viral infections. Here, we provide an overview of metabolomics methods including high-throughput analytical chemistry and multivariate data analysis to identify the specific metabolites associated with viral infections. This review also focuses on data interpretation and applications designed to improve our understanding of the pathogenesis of these viral diseases.

## 1. Introduction

Metabolomics, or the profiling of metabolites, is an omics type that aims to offer a comprehensive approach to identify, quantify, and characterize metabolites from biological systems. Metabolites usually refer to small molecules that are produced or modified by the biological metabolic processes of anabolism and catabolism. Detecting and appropriately analyzing metabolites can provide a functional readout of the cellular state when these are evaluated collectively or specifically. Metabolites provide directly detectable signatures of cellular biochemical activity, and are thus often less challenging to correlate with phenotypes than genes, which have functions that are subject to variable expression and epigenetic regulation, or proteins, which may also be regulated by complex chaperone systems or be post-translationally modified [1].

Metabolomics can be used along with genomics, transcriptomics, proteomics, lipidomics, and other omics methods to understand host−pathogen interactions at a molecular level [2]. Metabolomics is currently used in biomarker discovery as well as toxicology, personalized medicine, and drug discovery [3]. Diseases can cause specific changes to the metabolic profiles present in biological fluids and tissues before clinical symptoms manifest. Consequently, metabolomics has been used in both the prognostication and prediction of clinical outcome [4].

The study of viruses by metabolomics techniques is an excellent option. The study of the impact of viruses on metabolism during in vitro replication or infection in animal models or humans has produced original insights into these networks and introduced novel targets for therapy and biomarker development. The identification of common metabolic pathways utilized by viruses can reveal potential targets for broad-spectrum antiviral and vaccine strategies [5].

In addition to their applications in infectious disease diagnostics, metabolomics has been successfully been used for non-communicable diseases; for example, to identify inborn metabolic errors in newborns. The screening of newborn samples with mass spectrometry (MS) has become widely accepted [6]. However, relatively few metabolic biomarkers are currently being evaluated in large-scale clinical trials. A set of reliable predictive biomarkers would also be very helpful for the development of translational medicine initiatives related to drug discovery. In drug discovery and development, metabolomics can enable the identification of predictive biomarkers in vitro and in vivo, thus decreasing the risk in the drug approval process and facilitating clinical trial design [7]. Cancer has been an area of particularly intensive biomarker research. The use of liquid chromatography−mass spectrometry (LC-MS) and gas chromatography–mass spectrometry (GC-MS) to run metabolomics profiling of prostate cancer patient samples indicated several metabolic pathways that are altered, particularly including those supporting cell growth and proliferation, such as glycolysis, the tricarboxylic acid cycle, transcription and translation, and purine and pyrimidine metabolism [8]. Despite the extensive literature available for understanding the many aspects of viral pathogenesis and disease, the consequences of viral infections and the utility of metabolomics for the management of viral diseases remain highly challenging and generally lacking enough literature documentation to facilitate systematic reviews and meta-analyses [9,10,11]. In this qualitative scoping review, we describe the metabolomics approaches that are currently in both development and practical use that will facilitate the identification of common metabolic pathways in viral pathogenesis. We compare the clinical design, technical aspects, and statistical analyses of the published studies, found in PubMed and published up until 2023, with the purpose of identifying the most relevant biomarkers reported to date in viral pathogenesis by using metabolomics.

## 2. Metabolomics Analysis Workflow

A standard metabolomics analysis involves appropriate sample preparation, metabolite detection, data analysis, and interpretation. These methods have been widely reviewed previously and will be only briefly described below.

### 2.1. Biofluids Used in Metabolomics and Sample Preparation

A metabolomics workflow begins with the extraction and isolation of metabolites from biological samples. Previous reports have detailed several approaches for sample preparation involving homogenization, protein precipitation of samples, and comprehensive extraction methods [12,13,14]. A wide variety of biological matrices can be used in metabolomics studies, such as easily accessible biofluids (including plasma, whole blood, serum, urine, and saliva) and breath, as well as feces, organs, tissues, and cerebrospinal fluid (CSF), thus supporting their widespread use as diagnostic tools in clinical practice [15,16]. However, CSF is not suitable for large-scale screening, owing to its invasiveness, risk, and expense; thus, serum, plasma, and urine biomarkers are typically preferred [17].

### 2.2. Metabolomics Analytical Tools

Several technologies have been used to run metabolomics profiling in biological samples. Most of the metabolomics data produced to date have been generated using two main analytical techniques: nuclear magnetic resonance (NMR) and mass spectrometry (MS) [4]. Meanwhile these tools and the methods of using them are continually being improved, as are the analysis techniques for data interpretation.

NMR spectroscopy uses the properties of certain magnetic nuclei to measure the numbers and types of atoms in a molecule. For example, 1 H (“proton”) NMR spectroscopy can detect soluble hydrogen-containing molecules with a molecular weight of approximately 25 kDa or less, from proteins to small molecules. The resultant NMR data can also be multi-dimensional when homonuclear or heteronuclear experiments are operated, allowing for coupling with other 1 H, 13 C, and 15 N, and more magnetic nuclei. NMR spectra are subjected to pattern recognition analyses, in which complex multivariate and potentially overlapping data are simplified into two or three dimensions that can be readily understood and evaluated [18]. Clean or deconvoluted NMR spectra can be searched against available public, private, and/or proprietary databases for tentative metabolite identification.

MS is a technique that is used to measure and distinguish molecules on the basis of their molecular weight by the proxy of mass/charge (*m*/*z*) ratio of ionized species. This method requires three components: an ion source to generate ionized species, a mass analyzer, and a detector. Various types of each of these three components are suitable for different experiments. One MS analysis that can be performed with modern quadrupole instrumentation is tandem mass spectrometry (MS/MS), which is highly robust and sensitive, but has a lower mass resolution than other methods [19]. In contrast, a quadrupole linear ion trap (Q-TRAP)-MS provides fragmentations with a higher sensitivity and mass resolution than traditional quadrupoles, and is a more costly but generally superior form of tandem mass spectrometry [20]. The most common high-resolution MS is a hybrid quadrupole time of flight (QTOF)-MS, which has the advantage of fast simultaneous analyses of many compounds, as well as a high mass accuracy at the tradeoff of instrument expense and extremely large data sets [21]. For orbitrap-MS, the mass resolution, mass accuracy, and dynamic range are all high, the limit of detection is low, but the capital cost is accordingly expensive [22].

MS analysis is usually preceded by the chromatographic separation of molecules using liquid chromatography (LC) or gas chromatography (GC), depending on their physical properties. Different ionization methods will be operated in-line between the chromatography system and MS, such as electrospray ionization (ESI), atmosphere pressure chemical ionization (APCI), or atmospheric pressure photoionization (APPI) for LC-MS. LC-MS provides high metabolome coverage, and can be operated in normal phase LC for detecting polar metabolites and reverse-phase LC for detecting high hydrophobicity or relatively non-polar molecules. GC-MS, despite having somewhat lower coverage than LC-MS, is ideal for volatile compounds [23]. GC-MS typically offers a significantly superior chromatographic resolution of metabolites, and is most frequently operated using electronic ionization or “electronic impact ionization” (EI) although other ionization methods are available and used less often.

MS and NMR are each useful and complementary methods for conducting metabolomics studies, which must be selected appropriately based on the properties of the metabolites to be studied, the type of sample being evaluated, and the advantages and disadvantages of each analytical technique (Table 1) [24,25,26].

### 2.3. Metabolomics Approaches and Their Application

In metabolomics, two different approaches are used with different objectives: targeted and untargeted metabolomics. Both approaches can be used to identify biomarkers; however, several challenges remain before metabolomics can be widely applied into clinical research [27]. In general, targeted approaches analyze a relatively small and specific number of metabolites, typically as many as 20 metabolites at a time. These metabolites must have been chemically characterized before the start of data acquisition, and to select them would also typically require prior biochemical annotation with the established biological importance. However, targeted metabolomics approaches have significantly better selectivity and sensitivity than untargeted methods can offer [28]. This approach can be useful for pharmacokinetic (PK) studies of drug metabolism and for measuring the influence of therapeutics or genetic modifications on specific enzymes or proteins of interest that can be determined before the experimental design [29]. In contrast, the untargeted approach can more comprehensively analyze metabolites and reveal potentially unexpected changes, and is often used for conducting comparative metabolomics. Hundreds to thousands of metabolites from each sample can typically be measured in a single evaluation, although their identities may be unknown before the study, and even after. Due to the large volume and complexity of metabolomics data, high-performance bioinformatics tools are typically required for post-processing and analysis [30].

### 2.4. Statistical Analysis and Data Visualization

Metabolomics experiments lead to the generation of large and often complex data sets, which can include hundreds of metabolites or more per sample. The comprehensive evaluation of these outputs requires specialized data analysis that includes aspects of cheminformatics, bioinformatics, and statistics.

A data normalization step for raw data is generally required if the experimental design is to accurately quantify the features detected in the metabolomics analysis. Normalization can minimize undesirable systematic biases and background signals, thus yielding a modified data set that better highlights the relevant metabolite differences [31].

Differential analysis statistical methods are frequently used in untargeted metabolomics for biomarker discovery. Each feature or metabolite is considered to be a variable, and thus univariate and multivariate statistical tests can be used. Initial interrogation of this type of data typically is achieved using principal component analysis (PCA) for an unsupervised evaluation. Data visualization of PCA outputs is performed to identify outliers and common sample clusters. The differences in metabolite abundance can be presented, for example fold-change differences between samples and controls. The statistical significance of the differences is then measured by analysis of variance (ANOVA) and t-tests [32].

**Figure 1:**
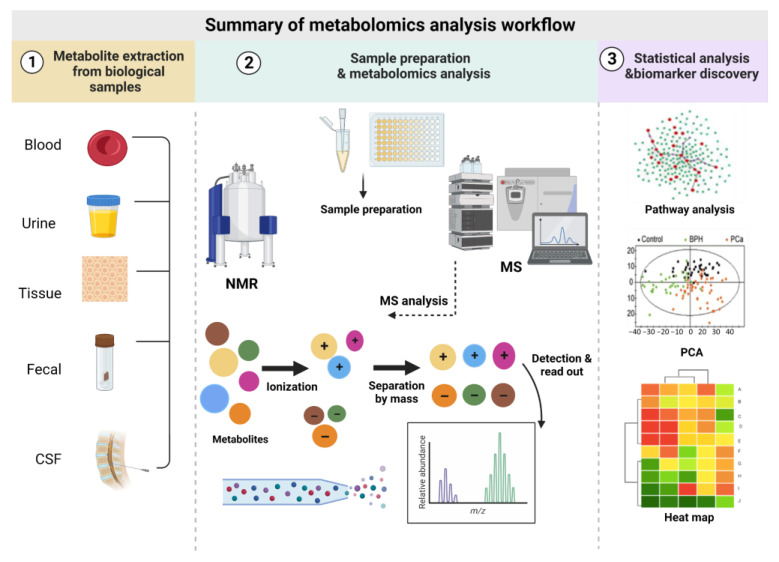
Summary of the metabolomics analysis workflow.

Both unsupervised and supervised multivariate tests can be performed. Supervised analyses, such as partial least squares regression discriminant analysis (PLS-DA) and orthogonal PLS-DA (OPLS-DA), can be used to generate models with groups assigned a priori. These types of models are usually used to classify the most important variables that can be used to separate predefined sample groups or can connect unclassified materials to samples with known class distributions (e.g., standards) for the purpose of making hypothetical or actual class assignments [33,34]. A summary of the metabolomics analysis workflow showed Figure 1.

## 3. Metabolomics Challenges

Metabolome complexity poses a major challenge in metabolomics studies. The organismal metabolome contains a wide variety of chemically diverse compounds such as lipids, organic acids, carbohydrates, amino acids, nucleotides, and steroids. The vast number of reported metabolites represent 52 different classes of compounds according to some metrics, resulting from the incorporation of a wide range of atoms, functional groups, and chemical structural moieties. In comparison, the building blocks of genes and proteins are relatively little. Genes comprise combinations of only four basic nucleotides, and proteins comprise combinations of 20 amino acids, and yet the order and spatial arrangement of these yield a huge number of identified genes and proteins with more left to be discovered.

The huge variability present in chemical structures results in a collection of analytes with markedly different physicochemical properties, such as polarity, solubility, and volatility. Furthermore, metabolites in the human body can be hard to evaluate because they are present in a broad and dynamic concentration range (picomolar to millimolar). A third hurdle is that not every metabolite is present in each tissue or biofluid, and they are certainly not expected to be present at the same concentration in different sample types. Finally, the organismal metabolome may be expected to contain exogenous metabolites, or xenobiotics, from food, medications, to environmental exposure, which may not be uniform among individuals. Therefore, performing comprehensive metabolomics is challenging. No single metabolomics method can measure the entire metabolome accurately [35].

## 4. Metabolomics Potential to Characterize Viral Infections

Wide varieties of disease-causing or pathogenic viruses infect humans, resulting in different clinical outcomes, ranging from mild and self-limiting to deadly and rampant [36]. Viral families such as Filoviridae, Arenaviridae, Bunyaviridae, Paramyxoviridae, Coronaviridae, Orthomyxoviridae, Flaviviridae, Togaviridae, and Hepeviridae are known to infect both humans and animals [37]. There have been many catastrophic viral disease pandemics caused by newly emerging and/or re-emerging viral pathogens, and these have resulted in millions of human deaths. For example, the “Spanish flu” influenza pandemic of 1918 was the most lethal pandemic in recent history, as over 50 million people died from the disease during the period of a few years. The subsequent emergence of additional influenza flu pandemics, such as the “Asian flu” and the “Hong Kong flu” in 1957 and 1968, respectively, resulted in about three million deaths [38]. Between 2002 and 2003, a novel severe acute respiratory syndrome coronavirus (SARS-CoV) disease emerged and infected 8098 people, leading to 774 deaths, according to the World Health Organization [39]. Although relatively few people died from this SARS outbreak, the mortality rate by percentage was quite high, and an international response was initiated to contain the spread. In 2009, a new strain of influenza A virus (IAV) H1N1 (H1N1pdm09) disease emerged, which killed an estimated 151,700–575,400 people worldwide during just the first year of circulation [40]. In 2013, a new strain of avian IAV (H7N9), known as “Bird Flu”, and Middle East respiratory syndrome (MERS)-CoV were both discovered [37]. Some viruses re-emerge after a period of time without notable outbreaks, and such was the case for Ebola virus (EBOV) in 2014 [41]. In addition, the resurgence of Zika virus (ZIKV) occurred between 2015 and 2016 [42]. The World Health Organization in 2015 issued new guidance for best practices in viral disease nomenclature to lessen secondary burdens from the incredible human health impact of these viral disease pandemics that had been observed because “certain disease names provoke a backlash against members of particular religious or ethnic communities, create unjustified barriers to travel, commerce and trade, and trigger needless slaughtering of food animals” [43]. From 2019 through to the present day, the ongoing corona virus disease 2019 (COVID-19) pandemic, caused by SARS-CoV-2, has ravaged many communities worldwide and infections are still rising globally. In the same time period, the re-emergence of EBOV occurred in parts of Africa and the Orthopoxvirus disease “monkeypox” (MPOX) pandemic has affected upwards of 100,000 people globally with over 98% of confirmed cases identified “in locations that have not historically reported mpox” according to the US CDC, such as the regions of the Americas and Europe.

The application of metabolomics to characterize infectious diseases is an emerging area of research and practice. Infectious diseases are typically diagnosed as follows: (1) directly via microscopy, culture, or viral diagnostic tests such as the antigen test or the nucleic acid amplification test (NAAT), or (2) through indirect methods such as antibody tests [44]. Other laboratory parameters are also used in the diagnosis of infectious diseases, such as blood cell count, erythrocyte sedimentation rate (ESR), detection of neutrophils at the site of infection, and levels of non-specific inflammatory biomarkers (e.g., C-reactive protein (CRP) and procalcitonin (PCT)). As a general principle, metabolomics approaches can be used to detect pathogens directly or via target specific host response biomarkers to identify signs of infection/disease progression [44]. A variety of viral infections have been studied using metabolomics by employing the earlier discussed methodologies such as NMR, GC-MS, or LC-MS, and using both targeted and untargeted approaches [45]. Metabolomics analyses can also be used to investigate and understand the pathogen–host interaction in a defined clinical perspective. In addition, specific disease states can have characteristic biomarker signatures that can be detected by metabolomics. As a result, validated biomarkers can be used for disease diagnosis, prognosis, and staging, as well as for the assessment of new preclinical and clinical therapeutic agents [25]. A growing number of published studies have outlined the use of metabolomics approaches to investigate viral infections. The following subsections of this scoping review focus on the viral infections that have been the subject of these metabolomics studies and are highly relevant to human health.

### 4.1. Metabolomics Study of Respiratory Pathogens

Viral respiratory infections can cause mild symptoms or severe morbidity and death, and potentially spread rapidly due to exposure while breathing. Some of these viruses circulate seasonally and result in recurring epidemics. The recent emergence of SARS-CoV-2 exemplifies the high infection rates that can be demonstrated by acute respiratory viruses [46]. Patients with severe respiratory viral infections are primarily hospitalized because they develop pulmonary inflammatory disorders, which often lead to lung tissue damage, edema, and further exacerbated inflammation [47]. The most common acute respiratory viruses are adenovirus (AdV), CoV, influenza virus (IV), parainfluenza virus (PIV), respiratory syncytial virus (RSV), and rhinovirus (RV). These viruses are all linked to the development of pneumonia, which causes a substantial burden of illness [48].

#### 4.1.1. Coronaviridae

CoVs belong to the order Nidovirales, family Coronoviridae, and subfamily Orthocoronaviridae. All known CoVs are of zoonotic origin and cause respiratory and intestinal infections in humans and other animals [49]. Three major CoV outbreaks have been reported since 2002, including SARS-CoV, MERS-CoV, and most recently SARS-CoV-2 [50]. COVID-19 is a highly contagious respiratory illness caused by SARS-CoV-2 [51]. According to the World Health Organization, over 762 million confirmed cases and 6.8 million confirmed deaths have been attributed to COVID-19 worldwide as of mid-April 2023. Meanwhile, many estimate significant undercounting due to unconfirmed or unreported cases. In the absence of effective and available treatments for the massive patient population, the ability to make a rapid and accurate diagnosis is crucial. Metabolomics is an excellent tool to use for the diagnosis, prognosis, and drug development to combat COVID-19. The advantages of this approach include: (1) the ability to generate vast amounts of comparable data; and (2) the rapid screening of molecules to identify biomarkers for the diagnosis and prediction of disease severity [25,44,52].

To date, several studies have used a metabolomics approach to determine factors such as disease severity, intensive care unit (ICU) prioritization, and various associated complications in patients with COVID-19. One of these studies showed that metabolic changes could be used to predict recovery patterns in critically ill SARS-CoV-2-infected patients [53]. In this study, a targeted metabolomics approach was used to analyze 39 serum samples taken from patients with COVID-19 within 48 h of hospital admission. The patients were subjected to invasive mechanical ventilation (IMV) in ICUs. Metabolites detected in the patients’ serum one week later, including kynurenine, 3-methylhistidine, ornithine, p-cresol sulfate, C24, and sphingomyelin, were determined to be accurate predictors of their IMV duration [53]. Despite several other metabolomics studies of COVID-19 [54,55,56], Elrayess et al. were the first to use metabolomics to show that patients with type 2 diabetes mellitus and hypertension were more likely to develop severe COVID-19 than patients without these conditions [57]. Targeted metabolomics using serum samples from patients with different COVID-19 severities, diabetes status, and hypertension status was performed using LC-MS/MS, followed by multivariate and univariate data analysis models [57]. The results showed that patients with diabetes and hypertension had more severe COVID-19 and lower levels of specific triacylglycerols, which are essential for regulating the inflammation response [57]. In a previous study, the untargeted NMR analysis of plasma-EDTA from 30 SARS-CoV-2-infected patients was used to identify the distinct metabolomics and lipidomics signature of COVID-19 [54]. The same approach was also used to assess tocilizumab treatment efficacy in a subset of patients with COVID-19, which resulted in partial reversion of the metabolic alterations induced by SARS-CoV-2 infection [54]. Thus, NMR-based metabolomics and lipidomics profiling have been demonstrated to provide novel insights into the pathophysiology of COVID-19 and the associated treatment outcomes.

In conclusion, there has been a significant amount of research on the use of metabolomics in studying coronavirus infections. More studies are needed to investigate the metabolic changes that occur during various stages of COVID-19 disease progression and how these changes relate to clinical outcomes.

#### 4.1.2. Orthomyxoviridae

The Orthomyxoviridae family of viruses includes four genera (Alphainfluenzavirus, Betainfluenzavirus, Gammainfluenzavirus, and Deltainfluenzavirus) containing IVs type A, B, C, and D, respectively, which are characterized by a segmented single-stranded negative-sense RNA genome. Specifically, influenza A virus harbors eight fragments encoding at least 11 proteins [58,59]. There have been several well-known pandemics of influenza IAV disease: the Spanish influenza (H1N1) in 1918, the Asian influenza (H2N2) in 1957, the Hong Kong influenza (H3N2) in 1968, avian influenza (HPAI) A(H5N1) in 2003, pandemic swine influenza H1N1 (pH1N1), and the Mexican influenza (H7N9) in 2018 [60,61]. A global epidemic of influenza causes a substantial amount of morbidity and mortality, in spite of the fact that several viral vaccines and inhibitors are available. This is because IVs have a high mutation rate and are constantly evolving, resulting in millions of new infections per year [62]. Metabolomics and lipidomics approaches have been used to understand the replication and pathogenesis mechanisms of IVs. In a recent study, non-targeted metabolic profiling was performed using LC-MS in combination with tandem MS, followed by multivariate analysis methods. Peripheral blood mononuclear cells (PBMCs) were spinoculated with Influenza IVs A produced using the Madin-Darby canine kidney cell culture system. The infected PBMCs underwent changes in their lipid, polyamine, catecholamine, and vitamin biosynthesis pathways [63]. The metabolism of immune cells was thus determined to explain the inflammation caused by IVs in that study. Niessen et al. reported using LC-MS with tandem MS, operated in selected-reaction monitoring (SRM) mode, to study small-molecule antiviral agents in the context of IV infections [60]. That study demonstrated that LC-MS-based pharmacokinetics studies of IVs treatment could be useful for therapeutic drug monitoring.

In conclusion, more comprehensive studies are needed to identify biomarkers and to better understand the metabolic pathways involved in influenza infection.

### 4.2. Metabolomics in Chronic Viral Infections

The term “chronic” is used to describe viral infections in which the pathogens cannot be cleared from the body. In these cases, the viruses persist in the infected individual, usually in association with specific cells or cell types. Latent, chronic, and slow infections are all types of persistent virus–host interactions with features that partially overlap one another [64]. One example of this type of infection is viral hepatitis, which is a significant public health challenge affecting millions of people in the world, with significant morbidity and mortality. Despite being taxonomically unrelated, the hepatitis A-E viruses are responsible for most of the viral hepatitis in the world. Among these, hepatitis B virus (HBV), hepatitis C virus (HCV), hepatitis D virus (HDV), and sometimes hepatitis E virus (HEV) infections may result in chronic disease. Several of the early metabolomics studies focused on chronic infections of HBV, HCV, human immunodeficiency virus (HIV), and human cytomegalovirus (HCMV) infections were performed using important methodologies that are summarized in this review [64,65,66,67].

#### 4.2.1. Human Immunodeficiency Virus (HIV)

HIV is a retrovirus that attacks and destroys the human immune cells. HIV specifically destroys white blood cells that express the CD4 cell surface antigen (i.e., CD4+ cells). This results in the loss of immunity, which leads to opportunistic infections, including tuberculosis, fungal infections, and severe bacterial infections, as well as some cancers [68]. The World Health Organization (WHO) estimates that there are approximately 38.4 million people living with HIV, with 2.7 million new infections having been reported in 2021 alone [69]. Despite the clear and inarguable success of combination antiretroviral therapy (cART), HIV remains an enormous public health problem throughout the world. Although cART and the availability of clinical testing have significantly increased the life expectancy of people living with HIV, medical treatment presents challenges at the individual level and existing clinical markers of disease progression remain unreliable [70]. Thus, it may be helpful to use metabolomics for identifying novel biological markers. A recent study [71] generated metabolic profiles for various underlying non-communicable diseases (NCDs) exhibited by individuals undergoing treatment for HIV infection. Untargeted metabolomics analysis was performed on samples collected from 87 HIV-negative (–) healthy (normal) normal controls (NCs), 87 HIV-positive (+) subjects with no known NCDs NCs, and 148 HIV+ subjects each with only one known with only one type of NCDs. Other diagnoses among individuals in these cohorts were included subclinical carotid atherosclerosis, neurological impairment, liver fibrosis, and neurological or renal impairment. Among the results of this study, all of the HIV+ participants exhibited viral suppression. Viruses and pathogenic bacteria can spread through the respiratory system with potentially life-threatening consequences.

Tuberculosis (TB) is caused by infection with the bacterial respiratory pathogen, Mycobacterium tuberculosis, and people living with HIV/AIDS (acquired immunodeficiency syndrome) are at high risk of contracting TB. TB remains a global burden and pandemic disease, and TB/HIV co-infections are becoming more prevalent as the number of new HIV cases grows throughout the world. While the nature of immunological deterioration associated with HIV infection has been well-established, the impact of these impairments on specific metabolomics profiles remains poorly understood. To address this information gap, Liebenberg et al. performed a study that used a metabolomics approach and compared the metabolites of one co-infected patient cohort with another. This research identified several metabolites that were differentially regulated specifically within immune cells, and the authors of the study concluded that the pathogenesis of HIV infection shared some features with TB infection, but was different in several other critical aspects [72]. Similarly, Hewer et al. [73] used a series of NMR-based metabolic profiles to distinguish serum samples from HIV-1-positive patients (both those who were and were not undergoing antiretroviral therapy) from those of HIV-1-negative controls. Among the findings of this study, the authors identified significant differences in lipid, glucose, and amino acid concentrations based on the NMR spectroscopic regions. These results were further supported by the study of Philippeos et al., which performed NMR metabolomics coupled with a logistic regression that revealed the same significant differences between the spectra of HIV-infected and HIV-uninfected individuals [74].

#### 4.2.2. Hepatitis B Virus (HBV)

Hepatitis B virus (HBV) is a partial strand DNA virus and a member of the Hepadnaviridae family. HBV infection can be either latent or chronic, depending on the nature of the host cell. Chronic HBV infection can lead to liver cirrhosis and ultimately hepatocellular carcinoma (HCC). In 2021, the WHO estimated that 12–25% of individuals diagnosed with chronic HBV infection will require treatment, depending on the setting and specific eligibility criteria [75]. Although an effective vaccine is available, HBV remains a major global health issue associated with high rates of morbidity and mortality. To date, there are still no drug regimens capable of curing HBV infection. The identification of typical disease-associated metabolomics signatures together with a patient’s entire metabolic phenotype may help to improve our current understanding of this disease and to identify the serum biomarkers that distinguish hepatocellular carcinoma (HCC) at an early stage from chronic hepatitis B (CHB) and liver cirrhosis [76,77].

Pan et al. used a LC-MS approach with both positive-ion and negative-ion modes to identify a series of serum biomarkers that distinguished early-stage HCC from CHB and liver cirrhosis [78]. In this study, progression from CHB to liver cirrhosis to HCC was accompanied by gradual decreases in serum levels of eicosapentaenoic acid, 5-hydroxy-6E,8Z,11Z,14Z,17Z-eicosapentaenoic acid, and glycyrrhizic acid [78]. The authors also identified metabolites that might contribute to a clinical diagnosis of HCC. Similarly, Yu et al. used an untargeted metabolomics approach combined with multiple analyses (e.g., PCA, PLS-DA, volcano plots, and pathway analysis) to evaluate the metabolic data analysis and to detect the metabolic effects of HBV replication on liver function and the progression of hepatic disease (hepatitis, cirrhosis, and liver cancer) [79]. Samples were collected from 199 patients with hepatic disease, including those with active and inactive HBV. The data analysis revealed strong differences in the extent of amino acid depletion and phosphatidylcholine biosynthesis that contributes to HBV replication [79]. The patient model featured in this study will require further validation by targeted metabolomics approaches designed to provide new insight into HBV pathogenesis and treatment [79]. Finally, Nguyen et al. analyzed metabolomics data collected at different disease stages from patients diagnosed with CHB. A combined analysis of the gut microbiome and metabolomics data revealed that ammonia detoxification, glutamine, and glutamate metabolism, and methionine metabolism pathways, as well as branched-chain amino acid imbalance and disorders of the tricarboxylic acid cycle were among the primary factors influencing the rate of CHB progression in patients with chronic disease [80].

#### 4.2.3. Hepatitis C Virus (HCV)

Hepatitis C virus (HCV) is a single-stranded RNA virus and a member of the Flaviviridae family. Similar to HBV, HCV infections can be acute or chronic. Furthermore, HCV is a major cause of advanced liver disease and can induce hepatocellular carcinoma and its many extrahepatic manifestations. Based on current WHO estimates, about 58 million people worldwide are infected with HCV, and approximately 1.5 million new infections emerge every year. Most new HCV infections appear to be the result of unsafe healthcare procedures and intravenous drug use [81]. While most genotypes of HCV respond effectively to direct-acting antivirals, some genotypes are less susceptible to these treatments [82]. Similar to the situation with other viruses, there are specific geographical differences in the distribution of HCV genotypes and the associated susceptibility of the infection to available drug treatment [81]. Hence, new antiviral strategies based on the metabolic characterization of HCV disease remain a primary goal. As one example, Shanmuganathan et al. compared the results of metabolomics profiling obtained via multisegment injection-capillary electrophoresis-MS with that resulting from NMR-based methods [83]. According to their findings, both techniques can quantify the serum metabolites both quickly and reliably for large-scale metabolomics studies with a high degree of consistency [83]. Thus, metabolomics can provide insight into HCV infection and its associated sequelae. Similarly, Fitian et al. performed a comprehensive metabolomics analysis using integrated and non-targeted metabolomics methodology using both GC/MS and UPLC-MS/MS to identify metabolic derangements in patients with HCV-associated HCC and cirrhosis [84]. Based on their findings, abnormal dicarboxylic acid metabolism, elevated bile acid metabolism, and elevated fibrinogen-cleaved peptides are all indicative of liver cirrhosis [84].

#### 4.2.4. Human Cytomegalovirus (HCMV)

Cytomegalovirus is an endemic and ubiquitous double-stranded DNA virus belonging to the Herpesviridae family. HCMV infections are typically detected in the salivary glands. Many individuals with HCM=V infection present with few to no clinical symptoms. Thus, this infection is diagnosed relatively rarely in the general population, despite the fact that it can be life-threatening in immunocompromised individuals. While HCMV has been studied and characterized extensively, we have only a minimal understanding of the effects of HCMV infection on global metabolism [85,86,87].

As evidenced by the current literature, metabolomics can be a useful tool for the study of HCMV-induced alterations in cell and tissue functions. One recent observational study designed to improve our understanding of HCMV infection in infants featured the use of 1 H NMR spectroscopy-based metabolomics approaches combined with multivariate statistical analysis [88]. The authors generated infant urine metabolic profiles to determine whether this approach might be feasible and useful in clinical settings. The 1 H NMR spectra were analyzed with PLS-DA, which revealed metabolic changes associated with HCMV infection that included increases in urine levels of alanine, betaine, dimethylamine, and glycine compared with the controls [88]. Polyethylene glycol was also detected and quantified in two samples, although this compound was determined to be most likely used as an intravenous vehicle for pharmacological treatment. In another study, Li et al. analyzed the plasma samples from infants who developed HCMV-associated liver damage using an untargeted GC/MS approach [89]. The findings were evaluated using OPLS-DA to identify the differences between HCMV-associated infantile hepatitis (HCMV-IH), infantile cholestatic hepatopathy (HCMV-ICH), extrahepatic biliary atresia (HCMV-EBHA), and samples from normal controls [89]. The analysis identified numerous potential biomarkers, including 29 differentially-detected metabolites associated with disorders of amino acid, fatty acid, and energy metabolism [89]. Five metabolites (carbamic acid, glutamate, L-aspartic acid, L-homoserine, and noradrenaline) were significantly overrepresented in samples from infants diagnosed with HCMV EHBA; this result suggests that the pathogenesis and outcomes of this disease differ from those associated with HCMV ICH [89]. Furthermore, these results provide a potential diagnostic tool that could be used to distinguish ICH from EHBA, as well as to explore the pathogenesis of HCMV-induced liver injury.

In conclusion, there is still a need for more comprehensive studies on the metabolic changes that occur during the various stages of HIV, HBV, HCV, and HCMV infection and how these changes are affected by comorbidities.

## 5. Metabolomics in Viral Neurological Infections

While most known viruses replicate in the peripheral tissue, some have developed unique strategies that facilitate their entrance into the nervous system where they can cause acute or persistent infections. Viral infections of the central nervous system (CNS, i.e., the brain and spinal cord) can disrupt neurological homeostasis and promote dysfunction, including serious, potentially life-threatening inflammation. In most cases, the CNS is protected from the sequelae of acute viral infections by the effective responses of innate and acquired immune cells, while others can elicit virus-induced immune-mediated CNS pathogenesis. Infections can range from mild to severe, and some can result in death [90,91,92]. Several groups have reported that respiratory virus infections may result in neurological symptoms. Examples of this phenomenon include human respiratory syncytial virus (RSV), IAV, coronaviruses (CoVs), human metapneumovirus (hMPV), and enteroviruses [93,94]. Viral CNS infections with a particular impact on infants include measles, mumps, rubella, and human parvovirus B19 [95,96]. Furthermore, rubella and parvovirus B19 can be transferred vertically from mothers to fetuses and may result in congenital infections [95,96].

There has been very little exploration of metabolomics being applied to studying viral infections of the CNS. Exploratory studies of the metabolomes of cerebrospinal fluid (CSF) in one or more disease states may provide a valuable window for assessing the effects of a given pathogen on metabolism within the CNS. These efforts might also lead to future targeted diagnostics and therapeutics of viral CNS infections. Related to this, there are numerous arboviruses and other viral causes of encephalomyelitis (EM) syndrome, including rabies. EM is the term used to describe inflammation of the brain (encephalitis) and spinal cord (myelitis) that frequently results in permanent disability. Clinicians face significant challenges when confronted with the need for a rapid and accurate diagnosis of EM. In an attempt to differentiate between these complex neurological infections, one recent study featured the use of 1 H NMR spectroscopy to analyze CSF samples from 27 patients with infections, which included Lyme disease, West Nile Virus meningoencephalitis, multiple sclerosis, rabies, and Histoplasma meningitis [97]. The results from these samples were compared with 25 controls. Cluster analyses permitted these samples to be distinguished based on infection status and, to a moderate degree, by the pathogen, with both shared and unique metabolic patterns observed in the various samples [97]. These preliminary results suggest that CSF metabolomics may eventually be used as a rapid screening test to enhance diagnostic accuracy and improve patient outcomes. Another study reported the first use of 1 H NMR spectroscopy as a means to differentiate between bacterial meningitis (BM), tuberculous meningitis (TBM), and viral meningitis (VM), the three most common forms of meningitis in children [98]. CSF samples from children diagnosed with BM (n = 85), TBM (n = 47), and VM (n = 35), as well as controls (n = 24) who were between the ages of 6 and 12 years of age, provided CSF samples over a 5-year period (1988–2003) [98]. Among the metabolites that were characterized, the authors reported differential detection of beta-hydroxybutyrate, lactate, alanine, acetate, acetone, acetoacetate, pyruvate, glutamine, citrate, creatine/creatinine, glucose, and urea [98]. Formal analysis revealed that the control group could be distinguished from the disease group with 96.4% accuracy; in contrast, the diagnosis of tuberculous meningitis achieved only 77.2% accuracy [98]. After the exclusion of cyclopropane levels, bacterial meningitis was classified correctly 84.4% of the time, while viral meningitis was classified correctly 83.3% of the time [98]. Taken together, the results suggest that the use of a combination of NMR spectroscopic data together with other routine clinical features may enhance the differential diagnosis of meningitis in children.

Patients with severe respiratory virus infections have also developed clinically-significant neurological manifestations. The most common respiratory viruses associated with these sequelae include IAV, parainfluenza viruses, RSV, adenoviruses, CoVs, enteroviruses, rhinoviruses, and hMPV. One recent study aimed to identify specific biomarkers of IAV-associated encephalopathy in infected patients. To address this question, serum samples were evaluated using capillary electrophoresis time-of-flight mass spectrometry (CE-TOF-MS). The analysis of these results identified five metabolites as potential biomarkers for IAV-associated encephalopathy and suggested that the tryptophan-kynurenine metabolic pathway might contribute to disease pathology [99].

In conclusion, limited studies have been conducted on viral neurological infections and metabolomics, and there is a need for more research in this area.

## 6. Conclusions and Future Perspectives

Despite the tremendous progress that has been made in recent decades, viral infections and their sequelae remain among the most challenging and demanding problems in current clinical practice. The COVID-19 era has shown that emerging viral infection can pose a serious health risk for the world, hence, there is an urgent need for improved viral metabolomic techniques. Likewise, while we now have a greater understanding of many aspects of viral pathogenesis and disease, many others have yet to be clarified. An improved understanding of the properties of viruses and their interactions with hosts will provide us with the tools that we need for the effective management of viral diseases. The metabolomics approaches described in this qualitative scoping review, it is important to acknowledge the limitations associated with the study, that are currently in both development, and practical use will facilitate the identification of common metabolic pathways used by viruses and the development of broad-spectrum antivirals and vaccines. The results highlighted in this qualitative scoping review suggest that metabolomics is a useful method for studying viral diseases, with plenty of future promise. The confirmation of many preliminary findings described in this review await future study. Summary of the main human metabolomics studies on virus infections. (Table S1). Abbreviations list (Table S2). 

## Figures and Tables

**Table 1:** Advantages and disadvantages of individual approaches.

Variable	NMR	MS
Sample preparation	No sample preparation or sample extraction	Extraction, desalting, filtration
Number of detectable metabolites	Tens of metabolites from a single spectrum collected at or above 600 MHz	Can detect hundreds of metabolites from a single chromatogram (based on whether GC-MS or LC-MS is used)
Sensitivity	Lower than MS (nanomolar); lack of sensitivity	Higher than NMR (picomolar)
Quantification	No standard is required; linear response	Standard required (isotope-labeled standard); matrix and ionization-dependent response
Repeatability/Reproducibility	Both techniques are highly precise and reproducible	
Instrument Cost	More expensive option and takes up more space than MS	Cheaper and occupies less space than NMR
Specific advantages	Non-destructive detection, good replication, and structure information	Sensitivity, a high number of detectable metabolites
Specific disadvantages	Low sensitivity and peak overlap	Ion depression effect, no structure information, and destructive detection

## Supplementary Materials

The following supporting information can be downloaded at: https://www.mdpi.com/article/10.3390/metabo13080948/s1. Table S1: Summary of the main human metabolomics studies on virus infections. Table S2: Abbreviations list.
