# Supplementary material for: Metabolomics Approaches for the Diagnosis, Treatment, and Better Disease Management of Viral Infections
Source: Metabolites. 2023 Aug 15;13(8):948. doi: 10.3390/metabo13080948 (PMC10456346; doi:10.3390/metabo13080948)
Supplement: Supplementary file 1 [file metabolites-13-00948-s001.zip › Table S1 Summary of the main human metabolomics studies on virus infections.pdf]

Table S1 . Summary of the main human metabolomics studies on virus infections

Metabolomics approaches	Method	Type of virus	Cohort/ Biofluid	Key metabolites	Biomarker association	Authors
Targeted	FIA–MS/MS	SARS-CoV-2	39 patients/ Serum	kynurenine, 3-methylhistidine, ornithine, p-cresol sulfate, C24, and sphingomyelin	Predictive biomarkers of intensive care unit and mechanical ventilation duration in critically ill for Coronavirus disease	Taleb et al. [1]
Targeted	FIA–MS/MS	SARS-CoV-2	115 patients/ Serum	palmitic , docosapentaenoic , docosahexaenoic and oleic acids	Associated with increased SARS-CoV-2 severity in diabetic and hypertensive patients, offering potential novel diagnostic and therapeutic targets.	Elrayess et al. [2]
Untargeted	^{1}H NMR	SARS-CoV-2	30 patients/ plasma	pyruvate ,3-hydroxybutyrate, citrate and free amino acids (alanine, glycine, glutamine, histidine)	Assess metabolites alterations due to SARS-CoV-2 infection and evaluate the response to tocilizumab treatment	Meoni et al. [3]
Untargeted	ESI-MS/MS	influenza	not mentioned/ PBMCs	monodehydroascorbate radical, L-gluconate, orotidine 5′-phosphate, CDP-ethanolamine, 3-dehydrosphinganine, Sphingosine, *S*-formylglutathione, Resolvin E1, 3,4-dihydroxymandelate, monodehydroascorbate, putrescine	Understand the replication and pathogenesis mechanisms of influenza	Karimi et al. [4]
Targeted	CE-TOF-MS	influenza	34 pediatric patients / Serum	succinic acid, undecanoic acid, kynurenine, decanoic acid, cysteine and quinolinic acid	Biomarker discovery to understand the pathophysiology of encephalopathy linked to influenza.	Torii et al. [5]

Untargeted	UPLC-MS and GC-TOF-MS	HIV	87 HIV-positive and 148 HIV+ NCDs/ Plasma	plasmalogen, phosphatidylcholines, phosphatidylethanolamines, and glutamine	Metabolites may serve as crucial targets for investigating the pathophysiology of non-communicable diseases (NCDs) in HIV infection and developing treatments	Ding et al.[6]
Untargeted	^{1}H NMR	HIV	34 HIV-1 positive patients, 29 patients were receiving combination antiretroviral therapy treatment and 5 patients had not been treated with antiretroviral agents before/ Serum	choline, lactate, glycerol glucose, valine, and alanine.	Differentiate between individuals with HIV-1 positive and receiving antiretroviral treatment, and HIV-1 negative	Hewer et al. [7]
Untargeted	HPLC-HESI MS/MS	HBV	30 CHB patients, 29 LC patients, and 30 HCC patients/ Serum	1,2-diacyl-3-*β*-D-galactosyl-*sn* glycerol, 5-hydroxy-6*E*,8Z,11*Z*,14*Z*,17*Z*-eicosapentaenoic acid, glycyrrhizic acid ,taurodeoxy cholic acid	Differentiate between CHB, LC, and HCC patients. This can aid in the early detection and treatment of patients with HCC	Pan et al. [8]
Untargeted	UHPLC-Q-TOF/MS	HBV	199 hepatic disease patients /Serum	alanine transaminase, L-lactate dehydrogenase , alkaline phosphatase , aspartate transaminase, and gamma-glutamyl transferase	Changes that occur in patients with HBV replication and compare the metabolic alterations in the progression of liver disease induced by HBV infection	Yu et al. [9]
Untargeted	^{1}H NMR	HBV	90 patients with CHB/ Serum	glutamine , glutamate , methionine and tricarboxylic acid	Changes during the transition from immune tolerance to immune clearance phase and display distinct metabolomic	Nguyen et al.[10]

					profiles at different stages of medical treatment	
Targeted	MSI-CE-MS and ^{1}H-NMR	HCV	20 patients with HCV and 14 / Serum	hypoxanthine, acetoacetate, hydroxybutyric acid, lactic acid and glucose	Differentiation of liver disease severity and progression of liver fibrosis	Shanmugan-athan et al. [11]
Targeted and Untargeted	GC-MS and UPLC-MS/MS	HCV	30 HCC patients, 27 hepatitis C cirrhosis disease controls and 30 healthy / Serum	12-hydroxyeicosa-tetraenoic acid (12-HETE), 15-HETE, sphingosine, γ-glutamyl, xanthine, serine, glycine, aspartate, acylcarnitines and dicarboxylic acids	Metabolites were found to be associated with the presence of HCC and correlated with cirrhosis	Fitian et al. [12]
Untargeted	^{1}H NMR	HCMV	31 patients of HCMV and 22 controls/ Urine	betaine , glycine, alanine, dimethylamine, succinate,1-methylnicotinamide, acetate, and oxoglutarate	Confirms the usefulness of metabolomics for studying HCMV infection in clinical applications	Frick et al. [13]
Untargeted	GC-MS	HCMV	127 patients with HCMV infantile hepatitis, HCMV ICH, and HCMV EHBA/ Plasma	carbamic acid, glutamate, L-aspartic acid, L-homoserine, and noradrenaline	Potential diagnostic tool that could be used to distinguish ICH from EHBA as well as to explore the pathogenesis of HCMV-induced liver injury	Li et al.[14]
Untargeted	^{1}H NMR	Arbovirus: West Nile Virus and rabies	27 patients with infections that included Lyme disease, West Nile Virus meningoencephalitis, multiple sclerosis, rabies, and Histoplasma meningitis and 25 controls/ CSF	ketones, pyruvate, carnitine, glycine, glutamate, 2-oxoglutarate, sobutyrate, fructose, *N*-acetylneuraminate, and serine	Used as a rapid screening test to enhance diagnostic accuracy and improve patient outcomes	French et al. [15]

Untargeted	^{1}H NMR	viral meningitis (VM)	children diagnosed with BM (n=85), TBM (n=47), and VM (n=35), as well as controls (n=24)/ CSF	beta-hydroxybutyrate, lactate, alanine, acetate, acetone, acetoacetate, pyruvate, glutamine, citrate, creatine/creatinine, glucose, and urea	The combined use of NMR spectroscopic data and other routine clinical features may improve the differential diagnosis of meningitis in children	Subramani-an et al.[16]

References

1. Taleb, S., H.M. Yassine, F.M. Benslimane, M.K. Smatti, S. Schuchardt, O. Albagha, A.A. Al-Thani, A. Ait Hssain, I. Diboun, and M.A. Elrayess, *Predictive Biomarkers of Intensive Care Unit and Mechanical Ventilation Duration in Critically-Ill Coronavirus Disease 2019 Patients.* Front Med (Lausanne), 2021. **8**: p. 733657.
2. Elrayess, M.A., F.S. Cyprian, A.M. Abdallah, M.M. Emara, I. Diboun, N. Anwardeen, S. Schuchardt, and H.M. Yassine, *Metabolic Signatures of Type 2 Diabetes Mellitus and Hypertension in COVID-19 Patients With Different Disease Severity.* Front Med (Lausanne), 2021. **8**: p. 788687.
3. Meoni, G., V. Ghini, L. Maggi, A. Vignoli, A. Mazzoni, L. Salvati, M. Capone, A. Vanni, L. Tenori, P. Fontanari, et al., *Metabolomic/lipidomic profiling of COVID-19 and individual response to tocilizumab.* PLoS Pathog, 2021. **17**(2): p. e1009243.
4. Karimi, Z., A.A. Oskouie, F. Rezaei, F. Ajaminejad, S.M. Marashi, and T.M. Azad, *The effect of influenza virus on the metabolism of peripheral blood mononuclear cells with a metabolomics approach.* J Med Virol, 2022. **94**(9): p. 4383-4392.
5. Torii, Y., Y. Kawano, H. Sato, K. Sasaki, T. Fujimori, J.-i. Kawada, O. Takikawa, C.K. Lim, G.J. Guillemin, Y. Ohashi, et al., *Quantitative metabolome profiling reveals the involvement of the kynurenine pathway in influenza-associated encephalopathy.* Metabolomics, 2016. **12**(5): p. 84.
6. Ding, Y., H. Lin, X. Chen, B. Zhu, X. Xu, X. Xu, W. Shen, M. Gao, and N. He, *Comprehensive metabolomics profiling reveals common metabolic alterations underlying the four major non-communicable diseases in treated HIV infection.* EBioMedicine, 2021. **71**: p. 103548.
7. Hewer, R., J. Vorster, F.E. Steffens, and D. Meyer, *Applying biofluid 1H NMR-based metabonomic techniques to distinguish between HIV-1 positive/AIDS patients on antiretroviral treatment and HIV-1 negative individuals.* J Pharm Biomed Anal, 2006. **41**(4): p. 1442-6.

8. Pan, H.Y., Q.Q. Wu, Q.Q. Yin, Y.N. Dai, Y.C. Huang, W. Zheng, T.C. Hui, M.J. Chen, M.S. Wang, J.J. Zhang, et al., *LC/MS-Based Global Metabolomic Identification of Serum Biomarkers Differentiating Hepatocellular Carcinoma from Chronic Hepatitis B and Liver Cirrhosis.* ACS Omega, 2021. **6**(2): p. 1160-1170.
9. Yu, L., Z. Zeng, H. Tan, Q. Feng, Q. Zhou, J. Hu, Y. Li, J. Wang, W. Yang, J. Feng, et al., *Significant metabolic alterations in patients with hepatitis B virus replication observed via serum untargeted metabolomics shed new light on hepatitis B virus infection.* J Drug Target, 2022. **30**(4): p. 442-449.
10. Nguyen, H.T.T., R. Wimmer, V.Q. Le, and H.B. Krarup, *Metabolic fingerprint of progression of chronic hepatitis B: changes in the metabolome and novel diagnostic possibilities.* Metabolomics, 2021. **17**(2): p. 16.
11. Shanmuganathan, M., M.O. Sarfaraz, Z. Kroezen, H. Philbrick, R. Poon, A. Don-Wauchope, M. Puglia, D. Wishart, and P. Britz-McKibbin, *A Cross-Platform Metabolomics Comparison Identifies Serum Metabolite Signatures of Liver Fibrosis Progression in Chronic Hepatitis C Patients.* Front Mol Biosci, 2021. **8**: p. 676349.
12. Fitian, A.I., D.R. Nelson, C. Liu, Y. Xu, M. Ararat, and R. Cabrera, *Integrated metabolomic profiling of hepatocellular carcinoma in hepatitis C cirrhosis through GC/MS and UPLC/MS-MS.* Liver Int, 2014. **34**(9): p. 1428-44.
13. Frick, M.A., I. Barba, M. Fenoy-Alejandre, P. Lopez-Lopez, F. Baquero-Artigao, P. Rodriguez-Molino, A. Noguera-Julian, M. Nicolas-Lopez, A. de la Fuente-Juarez, M.G. Codina-Grau, et al., *(1)H-NMR Urinary Metabolic Profile, A Promising Tool for the Management of Infants with Human Cytomegalovirus-Infection.* Metabolites, 2019. **9**(12).
14. Li, W.W., J.J. Shan, L.L. Lin, T. Xie, L.L. He, Y. Yang, and S.C. Wang, *Disturbance in Plasma Metabolic Profile in Different Types of Human Cytomegalovirus-Induced Liver Injury in Infants.* Sci Rep, 2017. **7**(1): p. 15696.
15. French, C.D., R.E. Willoughby, A. Pan, S.J. Wong, J.F. Foley, L.J. Wheat, J. Fernandez, R. Encarnacion, J.M. Ondrush, N. Fatteh, et al., *NMR metabolomics of cerebrospinal fluid differentiates inflammatory diseases of the central nervous system.* PLoS Negl Trop Dis, 2018. **12**(12): p. e0007045.
16. Subramanian, A., A. Gupta, S. Saxena, A. Gupta, R. Kumar, A. Nigam, R. Kumar, S.K. Mandal, and R. Roy, *Proton MR CSF analysis and a new software as predictors for the differentiation of meningitis in children.* NMR Biomed, 2005. **18**(4): p. 213-25.
