# Supplementary material for: Metabolomics Approaches for the Diagnosis, Treatment, and Better Disease Management of Viral Infections
Source: Metabolites. 2023 Aug 15;13(8):948. doi: 10.3390/metabo13080948 (PMC10456346; doi:10.3390/metabo13080948)
Supplement: Supplementary file 1 [file metabolites-13-00948-s001.zip › Table S2 Abbreviations list.pdf]

**Table S2**. Abbreviations list

| Abbreviation | Definition |
|---|---|
| AdV | Adenovirus |
| APCI | Atmosphere pressure chemical ionization |
| APPI | Atmospheric pressure photoionization |
| BM | Bacterial meningitis |
| cART | Combination antiretroviral therapy |
| CHB | Chronic hepatitis B |
| CRP | C-reactive protein |
| CSF | Cerebrospinal fluid |
| CNS | Central nervous system |
| EBOV | Ebola virus |
| EI | Electronic impact ionization |
| EM | Encephalomyelitis syndrome |
| ESI | Electrospray ionization |
| ESR | Erythrocyte sedimentation rate |
| GC-MS | Gas chromatography–mass spectrometry (GC-MS) |
| H1N1 | Spanish influenza |
| H2N2 | Asian influenza |
| H3N2 | Hong Kong influenza |
| HPAI | Avian influenza |
| H7N9 | Mexican influenza |
| HBV | Hepatitis B virus |
| HCV | Hepatitis C virus |
| HCC | Hepatocellular carcinoma |
| HCMV | Human cytomegalovirus |
| HCMV-IH | HCMV-associated infantile hepatitis |
| HCMV-ICH | HCMV-infantile cholestatic hepatopathy |
| HCMV-EBHA | HCMV-extrahepatic biliary atresia |
| HDV | Hepatitis D virus |
| HIV | Human immunodeficiency virus |
| HIV/AIDS | Acquired immunodeficiency syndrome |
| hMPV | Human metapneumovirus |
| ICU | Intensive care unit |
| IMV | Invasive mechanical ventilation |
| IV | Influenza virus |
| LC-MS | Liquid chromatography-mass spectrometry (LC-MS) |
| MERS-CoV | Middle East respiratory syndrome |
| MPOX: | Monkeypox |
| MS | Mass spectrometry |
| MS/MS | MS/MS : Tandem mass spectrometry |
| m/z | Mass/charge |
| NAAT | Nucleic acid amplification test |
| NCDs | Non-communicable diseases |
